# Pro-resolving mediators promote resolution in a human skin model of UV-killed *Escherichia coli*–driven acute inflammation

**DOI:** 10.1172/jci.insight.94463

**Published:** 2018-03-22

**Authors:** Madhur P. Motwani, Romain A. Colas, Marc J. George, Julia D. Flint, Jesmond Dalli, Angela Richard-Loendt, Roel P.H. De Maeyer, Charles N. Serhan, Derek W. Gilroy

**Affiliations:** 1 Centre for Clinical Pharmacology and Therapeutics, Division of Medicine, University College London, London, United Kingdom.; 2 Lipid Mediator Unit, Biochemical Pharmacology, William Harvey Research Institute, Bart’s and the London School of Medicine, Queen Mary University of London, London, United Kingdom.; 3 Division of Neuropathology and, Department of Neurodegenerative Disease, University College London Institute of Neurology, London, United Kingdom.; 4 Center for Experimental Therapeutics and Reperfusion Injury, Department of Anesthesiology, Perioperative, and Pain Medicine, Brigham and Women’s Hospital and Harvard Medical School, Boston, Massachusetts, USA.

**Keywords:** Inflammation, Eicosanoids

## Abstract

While the treatment of inflammatory disorders is generally based on inhibiting factors that drive onset of inflammation, these therapies can compromise healing (NSAIDs) or dampen immunity against infections (biologics). In search of new antiinflammatories, efforts have focused on harnessing endogenous pathways that drive resolution of inflammation for therapeutic gain. Identification of specialized pro-resolving mediators (SPMs) (lipoxins, resolvins, protectins, maresins) as effector molecules of resolution has shown promise in this regard. However, their action on inflammatory resolution in humans is unknown. Here, we demonstrate using a model of UV-killed *Escherichia coli*–triggered skin inflammation that SPMs are biosynthesized at the local site at the start of resolution, coinciding with the expression of receptors that transduce their actions. These include receptors for lipoxin A_4_ (ALX/FPR2), resolvin E1 (ChemR23), resolvin D2 (GPR18), and resolvin D1 (GPR32) that were differentially expressed on the endothelium and infiltrating leukocytes. Administering SPMs into the inflamed site 4 hours after bacterial injection caused a reduction in PMN numbers over the ensuing 6 hours, the phase of active resolution in this model. These results indicate that in humans, the appearance of SPMs and their receptors is associated with the beginning of inflammatory resolution and that their therapeutic supplementation enhanced the resolution response.

## Abstract 2

In humans, the appearance of specialized pro-resolving lipid mediators and their receptors is associated with the start of inflammatory resolution.

## Introduction

Inflammation is a protective response against infection or injury. When it becomes dysregulated as a consequence of genetic abnormalities (1, 2), the aging process (3), or environmental factors (4), our immune system has the capacity to cause extensive damage. Arthritis, asthma, and chronic obstructive pulmonary disease, while etiologically disparate, are examples of diseases unified by a dysregulated inflammatory response. The current strategy of treating inflammatory disease is based largely on inhibiting the factors that drive acute inflammation such as NSAIDs (5), steroids (6), and anti–TNF-α (7). Although these medicines ameliorate disease symptoms, they do not bring about a cure and are ineffective in a significant subset of patients. Furthermore, their side effects can hamper endogenous homeostatic systems, predisposing to infection. Thus, there is a need to identify more efficient and effective therapeutics, with one approach being to harness the body’s own resolution process for therapeutic gain (8).

In this regard, attention has turned to the other end of the inflammatory spectrum, resolution, to understand the endogenous processes involved in switching inflammation off. Our approach was to identify novel internal counterregulatory systems that terminate inflammation in order to provide new targets that can be manipulated pharmacologically to push ongoing inflammation down a pro-resolution pathway (9).

Consequently, resolution has now been extensively studied and conclusively demonstrated to be an active process with quantifiable indices and specific requirements (10). For instance, it has become apparent that for effective resolution to occur the inflammatory stimulus must be cleared (1, 2). Thereafter, proinflammatory cytokines/chemokines are catabolized (11, 12), while infiltrated effector granulocytes die, ideally by apoptosis (13), which allows for their nonphlogistic efferocytosis by cells of the monocyte/macrophage lineage (14). Alongside the delineation of these events was the identification of novel soluble mediators, and their receptors, that help these processes. While cyclopentenone prostaglandins were the first lipid mediators to be identified as being biosynthesized during and to bring about the resolution of acute inflammation (15, 16), additional families of novel resolution-phase lipid mediators derived from arachidonic acid (AA) as well as docosahexaenoic and eicosapentaenoic acid (DHA and EPA) have been identified (17). These specialised pro-resolving lipid mediators (SPMs) that include the lipoxins, resolvins, protectins, and maresins are produced via enzyme-mediated stereoselective conversion of these essential fatty acids to produce specific potent bioactive mediators (18–22).

Suitable human systems that lend themselves to delineating pro-resolution pathways and that are amenable to therapeutic intervention have been lacking (8). To address this, we recently characterized a novel model of resolution of inflammation triggered by intradermal injection of UV-killed *E*. *coli* (UVkEc) in forearms of healthy volunteers (23). This system allows detailed investigation of cells and soluble mediators that drive the different phases of inflammation. In addition, it allows for quantification of the vascular response of inflammation, a clinical marker not easily measurable in mouse models. More importantly, the use of skin also lends itself to the local administration of immune modulatory agents.

Using this model, we first carried out comprehensive lipid mediator profiling analysis of the inflammatory infiltrate during the critical phases of onset and transition to resolution. Here we found a temporal regulation of lipid mediator profiles during the course of this self-limited inflammatory response, with AA-derived eicosanoids being increased in the onset phase, while the omega-3–derived SPM appearance correlated with the resolution phase. In addition, the receptors that transduce the pro-resolution effects of these lipid mediators were identified on the microvascular endothelium and infiltrating leukocytes. Upon injection into the established inflamed site, SPMs exerted a pro-resolution impact as defined by enhanced PMN clearance during the resolution phase. Taken together, these results demonstrate for the first time to our knowledge that SPMs trigger a pro-resolution cascade in humans.

## Results

### Resolution checkpoints in UVkEc triggered acute inflammation.

**Figure 1:**
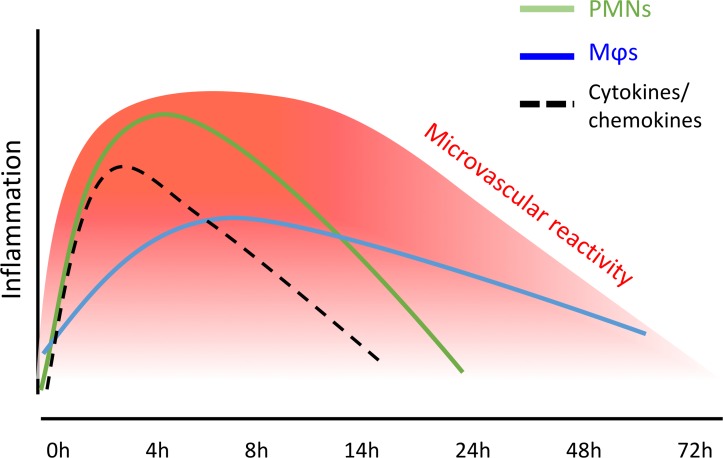
Summary of resolution markers in the UVkEc-triggered self-limiting acute inflammation model. Acute inflammation was triggered in the forearm of healthy volunteers by intradermal injection of 1.5 × 10^7^ UV-killed *E*. *coli* (UVkEc). Inflammatory exudate at the injection site was acquired into a suction blister. Temporal profile of inflammatory mediators as well as leukocytes in the exudate is depicted as published previously (23). MΦs, macrophages.

As recently demonstrated (23), intradermal injection of 1.5 × 10^7^ UVkEc elicits a self-resolving acute inflammatory response characterized by PMN infiltration as well as proinflammatory cytokine/chemokine production that peaks at 4 hours and begins to decline thereafter. Granulocyte clearance is followed by mononuclear phagocyte infiltration, with these cells increasing their expression of CD163, a typical marker of M2/pro-resolution macrophages. Vascular hyperemia increases with leukocyte infiltration and clearance, plateauing from 4 hours to 24 hours followed by a decline back to baseline levels by 48 hours; a summary of these temporal changes is presented in Figure 1.

Collectively, we demonstrated the critical requirements of resolution in this human model of inflammation, namely granulocyte clearance alongside proinflammatory mediator catabolism, followed by the acquisition of CD163 on phagocytosing macrophages; these events lead to resolution of the clinical sign of the response, vascular hyperactivity, by 48 hours.

### Resolution is preceded by generation of SPMs.

**Figure 2:**
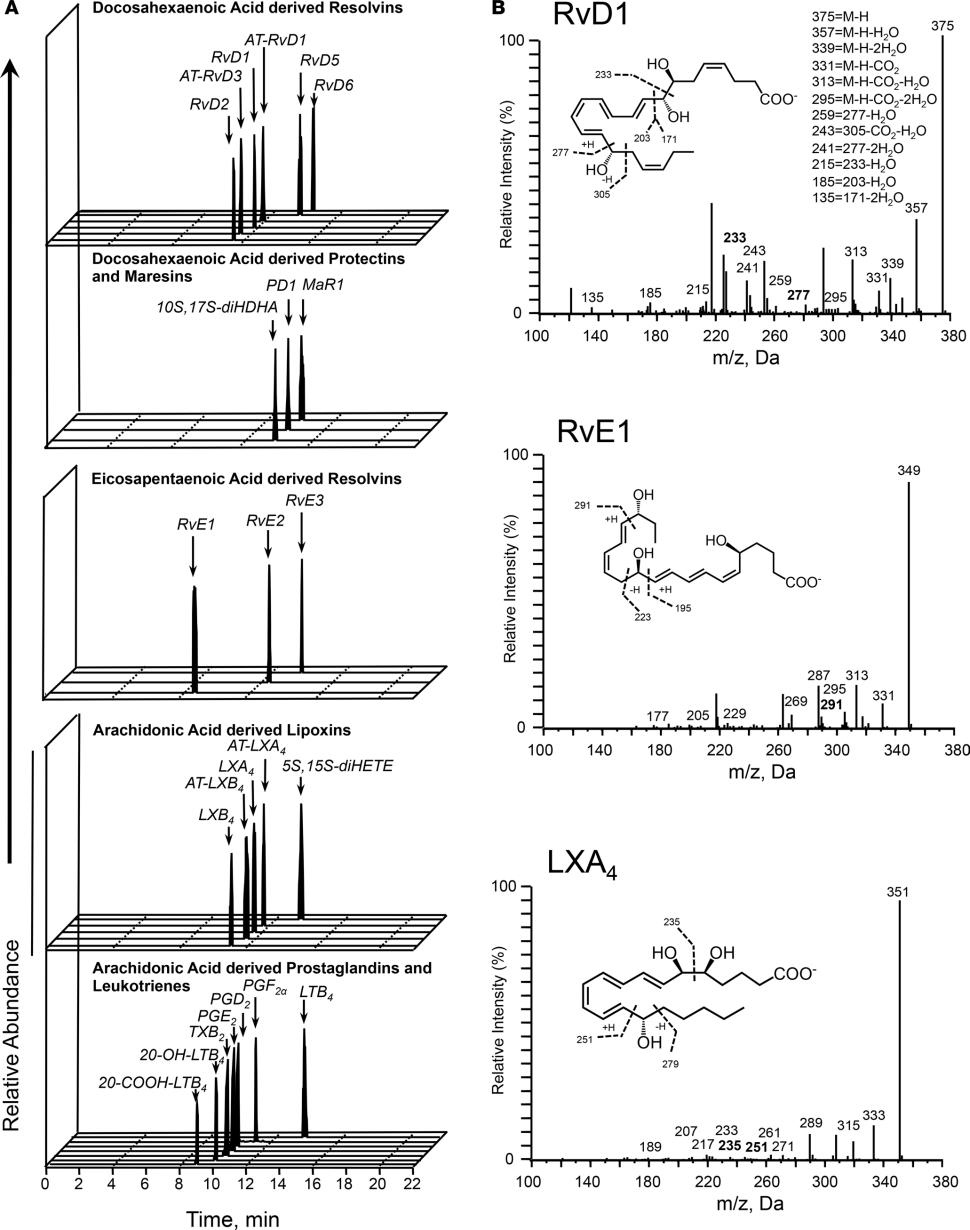
Human skin blister bioactive LM-SPM signature profiles. Acute inflammation was triggered in the forearm of healthy volunteers by intradermal injection of 1.5 × 10^7^ UV-killed *E*. *coli* (UVkEc). Inflammatory exudate at the injection site was acquired into a suction blister raised at 0, 4, 8, 14, and 24 hours. Infiltrated cells were centrifuged and cell-free supernatants were analyzed by lipid mediator (LM) metabololipidomics. (**A**) Representative multiple reaction monitoring (MRM) chromatograms for identified mediators in skin blister exudates. (**B**) Characteristic MS/MS fragmentation spectra employed in the identification of RvD1, LXA_4_, and MaR1. Results are representative of *n* = 34 exudates. Table 1 displays LM-SPM concentrations for the identified mediators. *n* = 6–7 donors per time point. Supplemental Figure 1 displays MS/MS spectra employed for the identification of mediators from the arachidonic acid, docosahexaenoic acid, and eicosapentaenoic acid bioactive metabolomes. SPM, specialized pro-resolving mediator.

**Figure 3:**
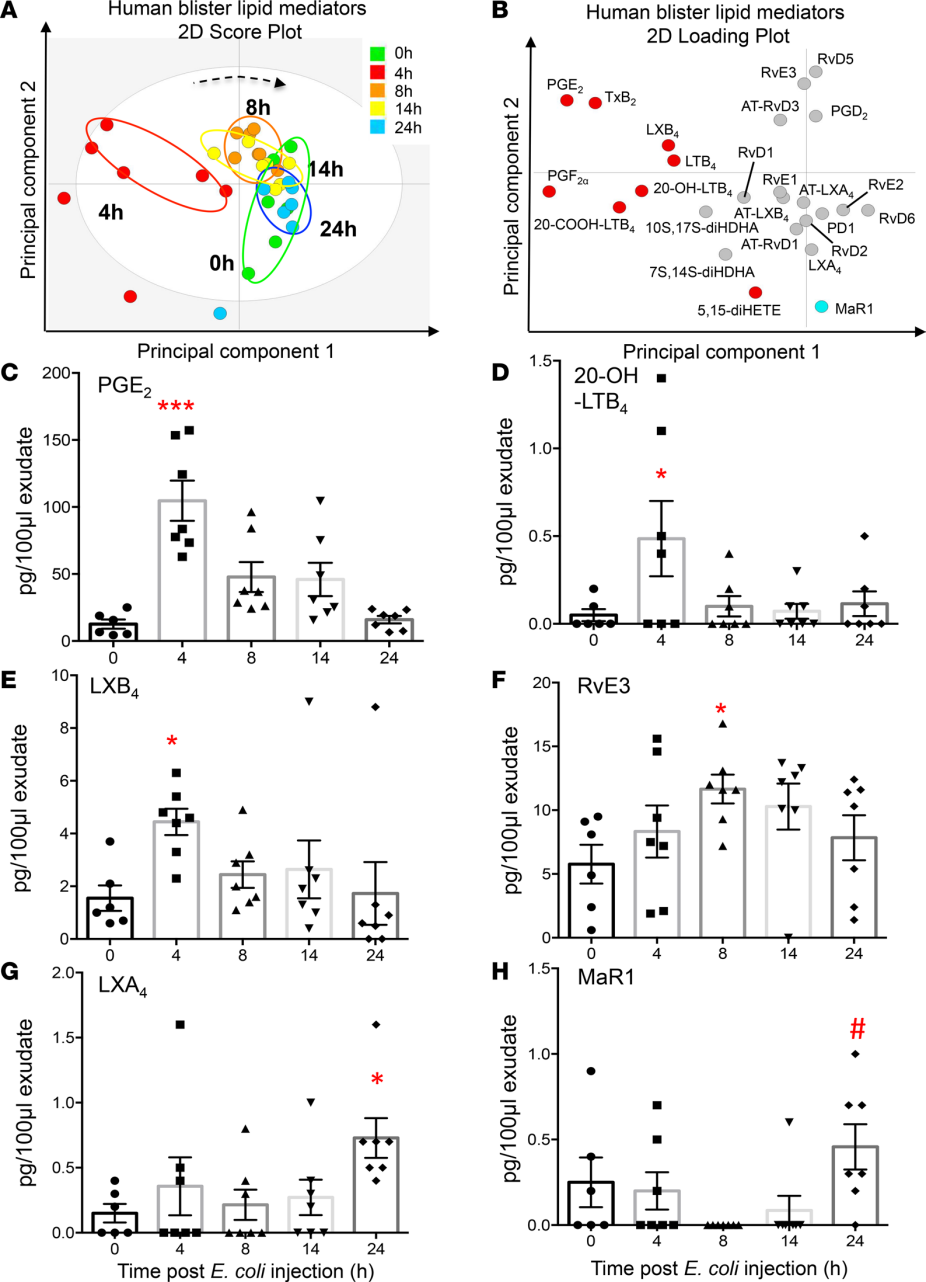
Temporal shift in LM-SPM concentrations in human skin blister exudates during inflammation-resolution. Acute inflammation was triggered in the forearm of healthy volunteers by intradermal injection of 1.5 × 10^7^ UV-killed *E*. *coli* (UVkEc). Inflammatory exudate at the injection site was acquired into a suction blister raised at 0, 4, 8, 14, and 24 hours. Cell-free exudates were obtained and lipid mediator identity and concentrations determined as in Figure 2. (**A**) Partial least squares-discriminant analysis 2-dimensional score plots of human skin blister LM-SPM exudates at 0, 4, 8, 14, and 24 hours. Gray ellipse denotes 95% confidence region. (**B**) 2-Dimensional score plot depicting the contribution of distinct mediators to the score plots. Concentrations for (**C**) PGE_2_, (**D**) 20-OH-LTB_4_, (**E**) LXB_4_, (**F**) RvE3, (**G**) LXA_4_, and (**H**) MaR1 at distinct intervals following UVkEc injection. Statistical comparison between groups was assessed by performing Kruskal-Wallis test and a post hoc Dunnet’s test to correct for multiple comparisons. *n* = 6–7 volunteers per interval. *P < 0.05 and ***P < 0.001 versus 0-hour time point; ^#^*P* < 0.05 versus 8-hour mediator levels. LM-SPM, lipid mediator/specialized pro-resolving mediator.

To investigate the temporal relationships in the regulation of lipid mediators at the site of inflammation we employed liquid chromatography–tandem mass spectrometry (LC-MS/MS) to obtain profiles for mediators from the AA, EPA, and DHA bioactive metabolomes (Figure 2A). In these exudates we identified mediators from all 3 essential fatty acid metabolomes including the cyclooxygenase-derived prostaglandin E_2_ (PGE_2_) and PGF_2α_ and the lipoxygenase-derived maresin 1 (MaR1), lipoxin (LX) A_4_, and resolvin (Rv) E1. These mediators were identified in accordance with published criteria that included matching retention time in liquid chromatography and at least 6 diagnostic ions in the MS/MS to authentic or synthetic standards (ref. 24 and Figure 2, A and B). To investigate the temporal regulation in these lipid mediator profiles we employed partial least squares discriminant analysis. This analysis identifies variables that contribute to the separation of the different time points based on lipid mediator levels. The score plot in Figure 3A illustrates the clustering of individual lipid mediator profiles based on similarity of overall lipid mediator levels. This plot indicates that lipid mediator profiles obtained from the 4-hour exudates were distinct from those obtained from exudate at 0, 8, 14, and 24 hours. In addition, we found a rightward shift in lipid mediator clusters from the 0-hour to the 24-hour clusters, suggesting a temporal regulation of lipid mediators. Assessment of the loading plot that provides an interpretation of the identified variables with the best discriminatory power, suggests that the eicosanoids PGE_2_, LTB_4_, the eicosanoid further metabolites, TxB_2_, 20-OH-LTB_4_ and 20-COOH-LTB_4_ together with the SPM LXB_4_ were mainly associated with the 4-hour cluster (Figure 3B). The loading plot also suggests that MaR1 was primarily associated the later phases of the inflammation-resolution course (Figure 3B).

Statistical analysis of lipid mediator levels at distinct intervals during the inflammation-resolution time course demonstrated that the levels of PGE_2_ and PGF_2α_ together with those of the TxA_2_ further metabolite TxB_2_ and the LTB_4_ further metabolites 20-OH-LTB_4_ and 22-COOH-LTB_4_ reach a maximum at the 4-hour interval and decrease subsequently (Figure 3, C and D and Table 1). At this interval we also found a significant increase in the SPM LXB_4_ (Figure 3E and Table 1). This was followed by a significant increase in the EPA-derived SPM RvE3 that reached a maximum at the 8-hour interval (Figure 3F and Table 1). In the latter stages of the resolution response we found significant increases in the AA-derived LXA_4_ (Figure 3G and Table 1). Of note, the macrophage-derived SPM MaR1 was found to decrease during the initial stages of the inflammatory response, reaching a minimum at the 8-hour interval; subsequently the concentrations of this SPM increased to levels that were significantly higher then those measured at the 8-hour interval (Figure 3H and Table 1). Therefore, these results identify a temporal shift in mediator levels, with inflammation-initiation eicosanoids reaching a maximum at the 4-hour interval that was followed by a temporal change in specific SPMs from all 3 bioactive metabolomes.

### SPM formation is associated with upregulation of SPM receptors.

**Figure 4:**
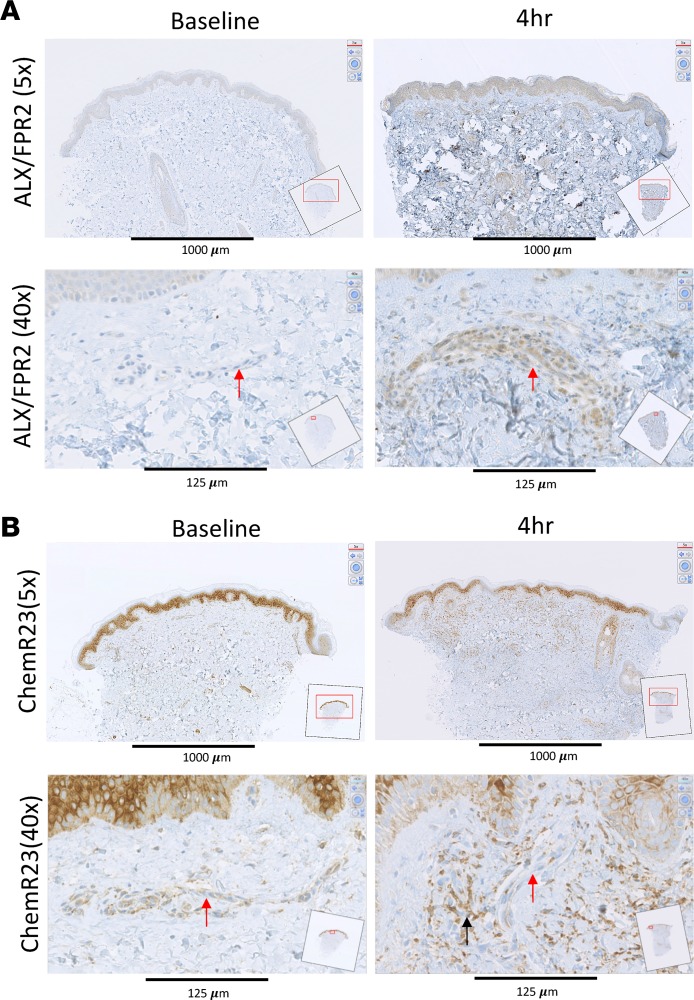
SPM receptors are differentially expressed on the endothelium and the infiltrating leukocytes — ALX/FPR2 and ChemR23. Acute inflammation was triggered in the ventral aspect of forearm of healthy volunteers by the intradermal injection of 1.5 × 10^7^ UV-killed *E*. *coli* (UVkEc) suspended in 100 μl of sterile saline. Four hours after injection, a 3-mm skin punch biopsy was taken from the inflamed site under local anesthesia. Naive skin was treated as baseline. Formalin-fixed paraffin-embedded skin sections were probed by immunohistochemistry for receptor identification. Low-magnification (×5) and high-magnification (×40) images at baseline and at the 4-hour time point are shown here for ALX/FPR2 (**A**) and ChemR23 (**B**). Red arrows highlight the endothelium and black arrow highlights the infiltrating leukocytes. Representative images from *n* = 3. SPM, specialized pro-resolving mediator.

**Figure 5:**
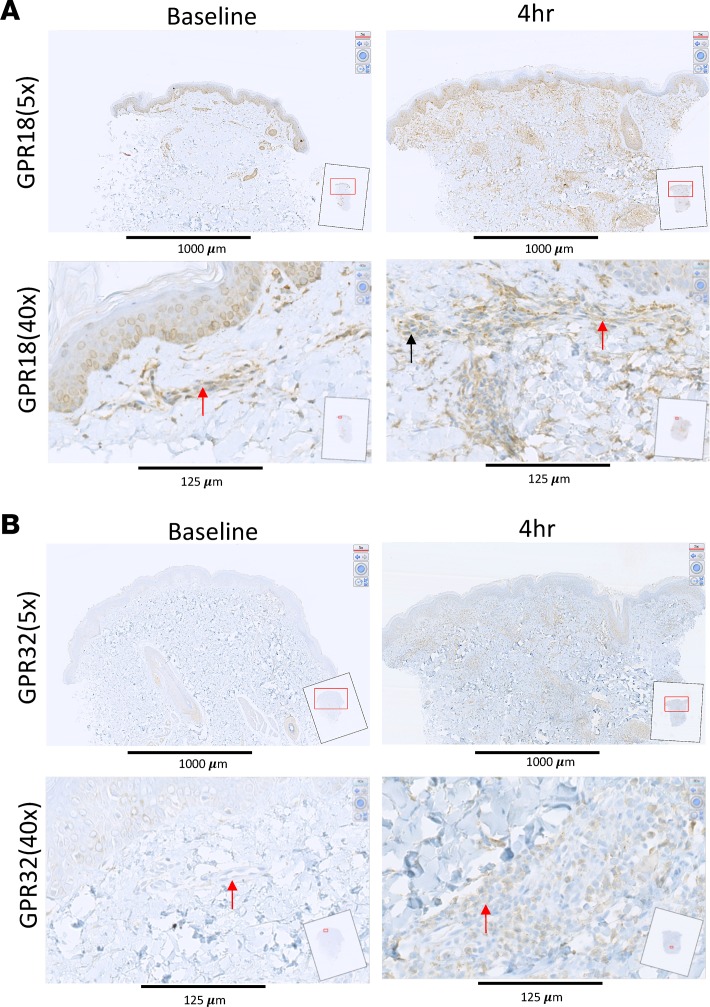
SPM receptors are differentially expressed on the endothelium and the infiltrating leukocytes — GPR18 and GPR32. Acute inflammation was triggered in the ventral aspect of forearms of healthy volunteers by the intradermal injection of 1.5 × 10^7^ UV-killed *E*. *coli* (UVkEc) suspended in 100 μl of sterile saline. Four hours after injection, a 3-mm skin punch biopsy was taken from the inflamed site under local anaesthesia. Naive skin was treated as baseline. Formalin-fixed paraffin-embedded skin sections were probed by immunohistochemistry for receptor identification. Low-magnification (×5) and high-magnification (×40) images at baseline and at the 4-hour time point are shown here for GPR18 (**A**) and GPR32 (**B**). Red arrows highlight the endothelium and the black arrow highlights the infiltrating leukocytes. Representative images from (*n* = 3). SPM, specialized pro-resolving mediator.

**Figure 6:**
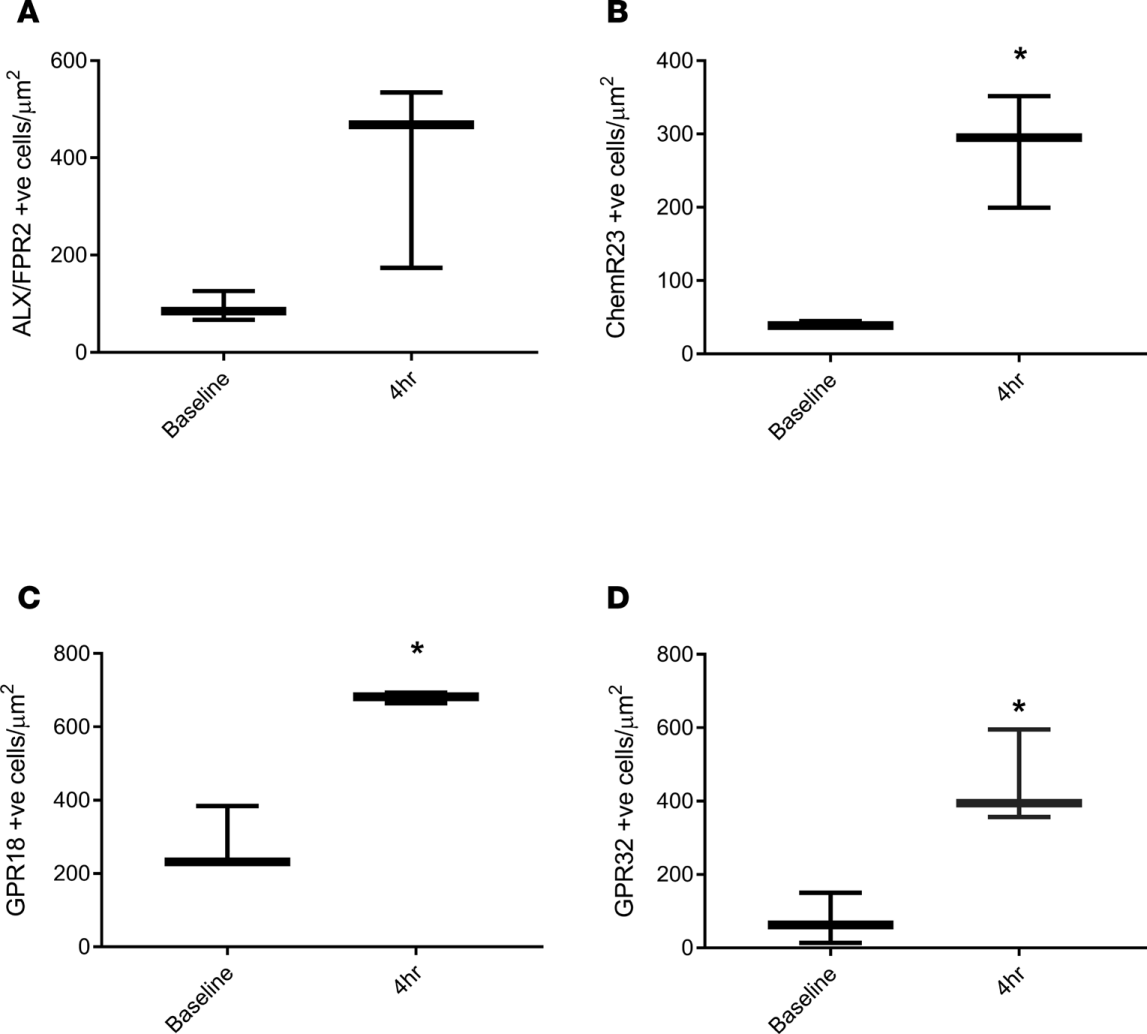
SPM receptor expression increases during acute inflammation. Acute inflammation was triggered in the ventral aspect of forearms of healthy volunteers by the intradermal injection of 1.5 × 10^7^ UV-killed *E*. *coli* (UVkEc) suspended in 100 μl of sterile saline. Four hours after injection, a 3-mm skin punch biopsy was taken from the inflamed site under local anaesthesia. Formalin-fixed paraffin-embedded skin sections were probed by immunohistochemistry for receptor identification, as shown in Figures 4 and 5. Increase in receptor expression from baseline to 4 hours following UVkEc injection for ALX/FPR2 (**A**), ChemR23 (**B**), GPR18 (**C**), and GPR32 (**D**) is shown here. Data expressed as box-and-whisker plots (box representing the median and the whiskers representing the maximum-minimum values). Statistical comparison between baseline and 4-hour expression was assessed by Wilcoxon’s matched-pairs test. *n* = 3 for each time point. *P < 0.05. SPM, specialized pro-resolving mediator.

We next determined the temporal and spatial expression of the known receptors for SPMs in naive (baseline) as well as 4-hour inflamed skin, which represent both the peak of the inflammatory response and the tipping point for its transition to resolution. UVkEc triggered ALX/FPR2 (receptor for AT-RvD1; see ref. 25) mainly on the endothelium with no detectable expression on endothelium in naive skin (Figure 4A). In contrast, while the receptor for RvE1 (ChemR23) (26) was found on endothelial cells in uninflamed skin, upon inflammation its expression was downregulated on endothelial cells but robustly upregulated on infiltrating leukocytes (Figure 4B). The receptor for RvD2, GPR18 (27), was expressed on the naive endothelium as well as infiltrating leukocytes of the inflamed skin, Figure 5A. The receptor for AT-RvD1 (human GPR32) (25), was absent on naive endothelial cells but was elevated on leukocytes in the inflamed skin (Figure 5B). Quantification of receptor expression is presented in Figure 6 where all 4 receptors are shown to be elevated at the site of inflammation following the injection of UVkEc.

### SPMs accelerate resolution of inflammation.

**Figure 7:**
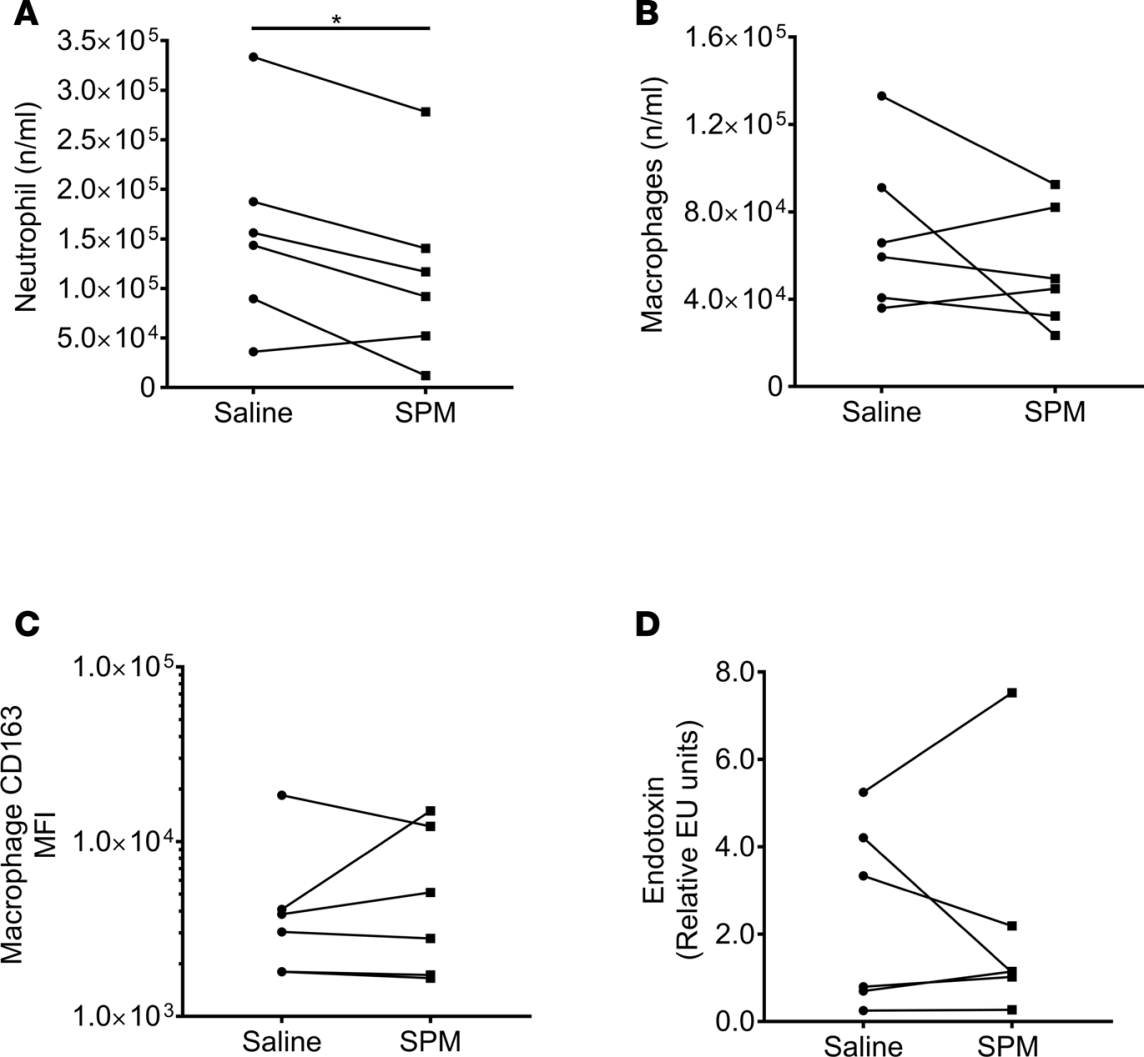
SPMs enhance neutrophil clearance. Specialized pro-resolving mediators (SPMs) (570 pM LXB_4_, 115 pM RvE1, 450 pM RvD2, 3.2 nM AT-RvD1 in 100 μl saline) were injected into the established inflamed site 4 hours after UV-killed *E*. *coli* (UVkEc), with an equivalent volume of saline injected into the contralateral forearm. Six hours later (or 10 hours after bacterial injection), a negative-pressure suction cup was placed over the inflamed site to acquire total inflammatory cells. Actions of SPMs on (**A**) PMN numbers were determined by polychromatic flow cytometry. Polychromatic flow cytometry was also used to determine the influence of SPMs on (**B**) macrophage numbers as well as (**C**) macrophage CD163 expression levels. Actions of SPMs on (**D**) endotoxin clearance 10 hours after UVkEc injection. Statistical comparison between groups was assessed by 2-tailed paired Student’s *t* test. Data are expressed as a before/after dot plot, *n* = 6/group. *P < 0.05.

We injected SPMs (570 pM LXB_4_, 115 pM RvE1, 450 pM RvD2, 3.2 nM AT-RvD1 in 100 μl of saline) into the inflamed site on one of the forearms 4 hours after UVkEc injection in order to study their potential impact on resolution of inflammation in vivo; the dose used reflected the highest concentrations present during inflammation-resolution (Table 1). The SPM panel selected consisted of members from each family (LX, E-series Rv, and D-series Rv) present within the blister exudate (Figure 2). In addition, chemical validation and purity of these SPMs are presented in Supplemental Figure 1 (supplemental material available online with this article; https://doi.org/10.1172/jci.insight.94463DS1). The impact of SPMs on inflammation/resolution was determined 6 hours later, i.e., 10 hours after UVkEC injection. The inflamed site on the contralateral forearm served as control and was injected with 100 μl saline only, 4 hours after UVkEc injection. The SPM-injected arm showed a significant decrease in total PMN numbers at 10 hours compared with the saline-injected arm (Figure 7A). SPMs did not affect macrophage numbers (Figure 7B) at the site nor their expression of CD163 (Figure 7C), a marker of enhanced efferocytosis. In addition, SPMs did not alter endotoxin clearance (Figure 7D) or indeed affect inflammatory cytokine levels at 10 hours (Supplemental Figure 2) or vascular hyper-reactivity, Supplemental Figure 3. These findings indicate that when injected therapeutically the mode of action of SPMs are essentially pro-resolution in nature.

## Discussion

Existing antiinflammatory therapeutics are aimed at inhibiting factors that drive inflammation including NSAIDs (PG) and biologics (TNF-α) (5–7). However, these are not effective in all patients, have undesirable side effects, and fail to switch off the underlying disease. As a result, much effort has focused on developing better treatments for diseases driven by chronic, ongoing inflammation. Furthermore, patients interact with their doctors once inflammation is already established, and so it makes sense to attempt to facilitate the resolution of this inflammation instead of only preventing further onset of inflammation. To this end, attention has now focused on how inflammation resolves, revealing that inasmuch as inflammatory onset is an active process, so also is resolution (28, 29). The sequential steps involved in this resolution process have been worked out, while some of the endogenous soluble mediators and receptors that drive these processes have also been elucidated (15, 26, 30–32).

The efficacy of SPMs has been demonstrated extensively in many different mouse models of inflammatory diseases and in human ex vivo assays (18–22, 33, 34). Indeed, multiple studies have validated SPM physiology in humans. For instance, SPMs have been identified in plasma of healthy volunteers (24) and their levels can be increased by n-3 fatty acid supplementation (35). SPMs have also been identified in patient populations from sites of inflammation, including in joint effusions of rheumatoid arthritis patients (31, 36), urine from COPD patients (37), and bronchoalveolar lavage of asthma patients (38). In addition, studies in sepsis patients have shown that SPM profiles in plasma can aid in stratification of disease severity and help in predicting their survival (39).

Taking this forward, we have now identified SPMs from all 3 essential fatty acid metabolomes during distinct phases of the inflammation-resolution process in a human model of acute self-limited inflammation triggered by UVkEc. Of note, identification of these mediators in both unchallenged and *E*. *coli*–challenged exudates is in line with several other published findings in both experimental systems (40, 41) and humans (41–44), which suggests that these mediators, in addition to orchestrating the termination of ongoing inflammation, are also important in maintaining tissue homeostasis. In addition, we determined the expression of the receptors that they work on, and have shown that exogenously adding these local mediators into the established site of inflammation hastens the resolution cascade. Specifically, SPMs, when added therapeutically at the resolution tipping point, led to reduced PMN numbers within the ensuing 6 hours. Taken together, these data show, for the first time to our knowledge, the potential efficacy of SPMs in humans. However, the mechanisms by which PMN numbers are reduced by SPMs are unclear at this stage. Given the lack of increased CD163 on resolution-phase macrophages belies increased efferocytosis while no reduction in classic PMN chemoattractants suggests that perhaps reversed transmigration back into systemic circulation (45) or enhanced lymphatic drainage might be an explanation for the pro-resolution effects of SPM in this human model. That notwithstanding, CD163 is one of many clearance receptors involved in efferocytosis whose expression might be upregulated by SPM and further work needs to be carried out to whether indeed SPMs enhance efferocytosis and by what mechanism or whether other mechanism such as reverse transmigration and/or enhanced lymphatic drainage are involved.

We found that ALX/FPR2 and GPR32 were both absent on the naive endothelium, while ChemR23 and GPR18 were expressed on these cells. Upon UVkEc injection, ALX/FPR2 expression was elevated on the endothelium, while GPR32, ChemR23, and GPR18 were increased on infiltrating leukocytes; levels of ChemR23 declined on the endothelium during inflammation. ChemR23 has been shown to dampen virus- as well as LPS-induced lung inflammation (46, 47), while also able to enhance microbial particle clearance and efferocytosis of apoptotic neutrophils by macrophages (48). Hence, it is likely that ChemR23 is upregulated on infiltrating cells in order to temper the severity of the inflammatory response and to facilitate resolution via bacterial and PMN clearance. GPR18, on the other hand, has been shown to be a receptor for anandamide as well as Δ^9^-tetrahydrocannabinol, the major psychoactive component of cannabis (49). Functionally, it may have a role in controlling the recruitment of CD8^+^ T cells to sites of inflammation (50) or, via its endogenous ligands, further amplify the pro-resolution response by triggering LXA_4_ and cyclopentenone PG biosynthesis (51). GPR32, the receptor for RvD1, is expressed on neutrophils, macrophages, and epithelial cells (31, 52, 53) where it transduces myriad pro-resolution properties of D-series resolvins. Finally, ALX/FPR2 is a receptor for annexin 1 and LXA4 with a role in controlling phagocyte trafficking and phagocytosis (54). However, in this human model of acute inflammation its expression was detected on inflamed endothelium only and was barely detectable on infiltrating leukocytes; of note, we used inflamed human appendix as a positive control for all of the above antibodies (data not shown). This was a surprising finding and certainly warrants further investigation, but may reflect some new role in regulating leukocyte trafficking into sites of inflammation. Indeed, the factors that regulate the differential upregulation and downregulation of these receptors between homeostasis and inflammation also warrant further investigation. Collectively, this model has lent itself to the identification of key internal receptors and signaling pathways central to controlling the evolution of acute inflammation and bring about its resolution and consequently provides the opportunity to identifying additional pathways important in host defense.

One of the principle concerns of exerting a pro-resolution effect in the context of live infection is that the host immune system might not efficiently clear the bacteria. The latter could arguably proliferate, resulting in continuous activation of the innate immune response leading to systemic or chronic inflammation. Indeed, this is very well exemplified in chronic granulomatous disease, which results from a failure of the phagocytic NADPH oxidase enzyme system to produce superoxide and kill invading infections leading to a predisposition to recurrent bacterial and fungal infections and the development of inflammatory granulomas (1, 2). However, in this model we have shown no increase in endotoxin levels to suggest failure of bacterial clearance as a result of supplementing SPMs. Indeed, SPMs efficiently clear PMNs as well as bacteria, as measured by a reduction in inflammatory site endotoxin levels in other models. While counterintuitive, this was demonstrated in a mouse model of *E*. *coli* infection. Here, SPMs given together with an antibiotic, ciprofloxacin, hastened resolution indices, enhanced phagocyte containment as well as killing of *E*. *coli*, and ameliorated the clinical signs of systemic inflammation (55). Using human macrophages, both RvD1 and RvD5 stimulated phagocytosis of *E*. *coli* in a GPR32-dependent manner (56). SPM also enhanced antibiotic effectiveness in clearing Gram-positive *Staphylococcus aureus* skin infections (56). Collectively, these findings indicate that SPMs not only stimulate pro-resolution processes in humans, but that they may also simultaneously enhance host immunity.

Another important point to emphasise is the use of a self-resolving model of acute inflammation to determine immune-modulatory properties of drug interventions. The logic behind this is manyfold, including the need to translate findings originally made in rodents to humans in terms of novel internal immune pathways. In this case, we wished to show that SPMs were synthesized at sites of inflammation in humans, that the receptors upon which they transduce their effects are expressed at the same time when their ligands are synthesized, and that when added back into this dynamic system are immune-modulatory. It transpired that the main effect recorded was a reduction in PMN numbers by SPMs. On this matter, there are a number of sequential events required to trigger the resolution cascade, one of which is PMN clearance (8). Certainly, and as alluded to above, PMNs are beneficial cells of the host immune response. However, their persistence at sites of infection and/or injury is also implicated in the pathogenesis of many chronic inflammatory diseases. Hence, that SPMs can reduce their numbers in inflamed tissues bodes well for their potential use to treat some diseases driven by ongoing inflammation.

In summary, we identified local SPM biosynthesis, as well as the receptors that transduce their actions, at the transition of inflammatory onset to its resolution in a human model of inflammation. In addition, we report that when added in a therapeutic manner, SPMs trigger resolution in this model, reducing PMN infiltration. This study is the first of its kind to our knowledge to show that harnessing the body’s own immune counterregulatory processes can be utilized for therapeutic gain.

## Methods

### Injection with UVkEc and acquisition of inflammatory exudate.

An intradermal UVkEc injection was used to induce an acute, resolving immune response, and the local site of inflammation was interrogated using a suction blister as described previously (23). Briefly, 1.5 × 10^7^ UVkEc in 100 μl of saline were injected intradermally into the forearms of volunteers. The inflammatory exudate from the injection site was obtained using a suction blister at specified time points. The blister exudate was centrifuged to separate cells, which were analyzed by flow cytometry. Cell-free exudate was stored at –80°C until further use. A detailed description of the preparation of UVkEc, intradermal injection, suction blister technique, and exudate collection and processing can be found in Motwani et al. (23).

### Lipid mediator profiling.

Blister exudates were placed in 2 volumes of ice-cold methanol containing deuterium-labeled standards (Cayman Chemicals). These were then kept at –20°C for 45 minutes to allow for protein precipitation and lipid mediators were extracted using C-18–based solid phase extraction as previously described (24). Methyl formate fractions were brought to dryness using a TurboVap LP (Biotage) and products suspended in water/methanol (50:50 vol/vol) for LC-MS/MS–based profiling. A Shimadzu LC-20AD HPLC and a Shimadzu SIL-20AC autoinjector, paired with a QTrap 5500 (ABSciex) were utilized and operated as previously described (24). To monitor each lipid mediator and deuterium-labeled internal standard, a multiple reaction monitoring (MRM) method was developed using parent ions and characteristic diagnostic ion fragments as previously described (24). This was coupled to an information-dependent acquisition and an enhanced product ion scan. Identification criteria included matching retention time to synthetic standards and at least 6 diagnostic ions in the MS/MS spectrum for each molecule. Calibration curves were obtained for each molecule using authentic compound mixtures and deuterium-labeled lipid mediator at 0.78, 1.56, 3.12, 6.25, 12.5, 25, 50, 100, and 200 pg. Linear calibration curves were obtained for each lipid mediator, which gave *r*^2^ values of 0.98–0.99.

### Lipid mediator validation.

LXB_4_, RvE1, RvD2, and AT-RvD1, purchased from Cayman Chemicals, were validated by assessing the characteristic UV chromophores for each of these mediators as well as their physical properties in LC-MS/MS, including characteristic MS/MS spectra (24). Lipid mediator concentrations were determined by using the extinction coefficient characteristic for each of the conjugated-double-bond systems as well as measuring maximal UV absorbance. Endotoxin content was assessed using ToxinSensor Chromogenic LAL Endotoxin Assay Kit (Antibodies Online), which was found to be below limits of detection in all preparations.

### Immunohistochemistry.

A skin punch biopsy (3-mm diameter) was obtained from the site of UVkEc injection at the specified time points. Baseline biopsies were obtained from the noninjected forearm of volunteers. Biopsies were immediately transferred to neutral buffered formalin for fixation. Formalin-fixed skin biopsies were embedded in paraffin wax and cut to a thickness of 4 μm. Skin sections were collected on glass slides and stained with antibodies after unmasking of antigen. The antibodies used in this study are as follows: rabbit anti–human FPR2 (Novus Biologicals, catalog NLS1878), rabbit anti–human ChemR23 (Thermo Fisher Scientific, catalog PA5-33438), rabbit anti–human GPR32 (Thermo Fisher Scientific, catalog PA5-33694), and rabbit anti–human GPR18 (LifeSpan Biosciences, catalog LS-A94). Immunohistochemistry was performed using the Ventana Discovery XT instrument (Ventana Medical Systems Inc.), using biotinylated secondary antibodies (swine anti-rabbit, Dako) and the Ventana DAB Map Kit for detection. The slides were hematoxylin counterstained. Images of the stained slides were obtained on a NanoZoomer (Hamamatsu).

### Quantification of immunohistochemical staining.

For quantification of staining, digital image analysis was performed using Definiens Developer 2.6 (Munich) at the University College London Institute of Neurology. The analysis comprised 3 phases: tissue identification, manual region selection, and stain detection. Tissue identification was performed at resolution equivalent to ×10 magnification. A composite raster image was produced containing the lowest (darkest) pixel value from the 3 color channels (RGB). A filtered version of this darkest image was then generated using a sliding window of 25 × 25 pixels (mean pixel value assigned to central pixel). The 99th centile, the threshold that separates the highest 1% of pixels from the lowest 99%, was calculated and adjusted by –15 (8-bit 256-color images) to give initial separation of tissue and background. The 10th centile in the filtered darkest image was then calculated for the background region and adjusted by –10, and all tissue with a higher value than this was reclassified as background. Small areas of tissue and background (<50 μm^2^) were removed, and then areas of tissue with area less than 1% of the total area of tissue were removed. The remaining area of tissue was used in subsequent analysis stages. Manual region selection was performed to identify 4 regions of interest within the identified tissue in each sample. Stain detection was performed at resolution equivalent to ×20 magnification. Identification of staining is based on the transformation of the RGB color model to an HSD representation (57). This provides a raster image of the intensity of each color of interest (brown and blue). Subtraction of the blue stain from the brown stain intensity at each pixel gives a third raster image, brown+ve, with a positive number where brown stain is prevalent. A fourth image is produced from 2 filtered layers: blue filtered with a sliding window of 3 and of 101. By assigning the value of blue (i.e., 101) subtracted from blue (i.e., 3) where the value is greater than 0.05 AU, a normalized level of blue staining is produced where the subtraction of the background level of blue stain enhances the real peaks in staining representing nuclei.

Nuclei were identified as areas greater than 10 μm^2^ with normalized blue greater than 0.1 AU. These were then further processed to separate merged nuclei. Any large nuclear shapes were excluded as artifacts. All remaining nuclei were surrounded by cell body to a depth of 7 pixels (0.45 μm/pixel). All pixels with brown+ve greater than 0.1 were classified as DAB stain, any cell body with greater than 20% DAB coverage was classified as positive cell body, and any nucleus with greater than 40% DAB coverage was classified as positive nucleus. Any cell containing a positive nucleus and/or positive cell body was classified as a positive cell. The average number of positive cells over the 4 regions of interest is expressed here as positive cells per square micrometer of tissue.

### Endotoxin measurement.

Endotoxin was measured using the limulus amebocyte lysate (LAL) Pyrogent 5000 kit. Blister fluid was pretreated by diluting in Tween-20 buffer followed by incubating at 70°C for 15 minutes as described previously for biological matrices (58).

### Laser doppler imaging.

Inflammation-induced microvascular hyperaemia at the site of injection was quantified by Laser Doppler Imager (Moor Instruments). The principle and procedure are described in detail in Motwani et al. (23). Briefly, the laser beam scanned the inflamed area over each forearm. The intensity of redness was reflected in the flux of red blood cells, and expressed in real time in a standardized color-coded image, which was then analyzed by moorLDI software.

### Flow cytometry.

Blister cells were suspended in 100 μl of cell staining buffer (PBS with 5% FCS, 0.1% sodium azide) and incubated for 30 minutes on ice with the following antibodies (all from Biolegend): CD3-FITC (clone HIT3a), CD14-BV605 (M5E2), CD16-APC (3G8), CD19-FITC (HIB19), CD56-FITC (HCD56), CD62L-PE-Cy7 (MEL-14), CD163-BV421 (M80), HLA-DR-BV510 (L243), and Siglec-8-PE (7C9). The stained cell sample was washed in PBS to remove excess antibody and then fixed in 1% paraformaldehyde. The fixed sample was acquired on a BD LSRFortessa within 4 hours. Flow cytometry data were analyzed by FlowJo software (Tree Star Inc.).

### Multiplex ELISA.

A human Proinflammatory Panel 1 mutiplex ELISA kit (Meso Scale Delivery) was used to measure cytokines. The cell-free blister exudate was diluted in appropriate assay diluent and the assay was performed as per the manufacturer’s instructions.

### Statistics.

GraphPad Prism software (version 7) was used for preparing figures and for statistical analysis. Differences between 2 groups were tested for statistical significance by 2-tailed Student’s *t* test (for paired normally distributed data). For more than 2 groups, differences were detected by Kruskal-Wallis test (for unpaired non–normally distributed data) followed by Dunnet’s test to correct for multiple comparisons. A *P* value less than 0.05 was taken as the threshold for significance.

### Study approval and study participants.

The study procedures were approved by the University College London Institutional Ethics Committee (UCL ID: 5051/001). Written informed consent was obtained from all volunteers. Healthy, young (18–50 years old), male, nonsmoking volunteers were recruited. Volunteers were excluded if they had a history of chronic inflammatory disease, allergies, recent illness (<1 month), vaccination within the last 3 months, were taking regular medication, or had taken any medication in the preceding week. Over the study duration, volunteers were asked to refrain from alcohol and heavy exercise.

## Author contributions

DWG and MPM designed the study. MPM, RAC, ARL, JDF, MJG, and RPHDM performed the research. MPM, DWG, RAC, JD, and CNS analyzed the data. JD and CNS contributed new reagents and analytical tools. DWG wrote the paper with critical input from all co-authors.

## Supplementary Material

Supplemental data

## Figures and Tables

**Table 1:**
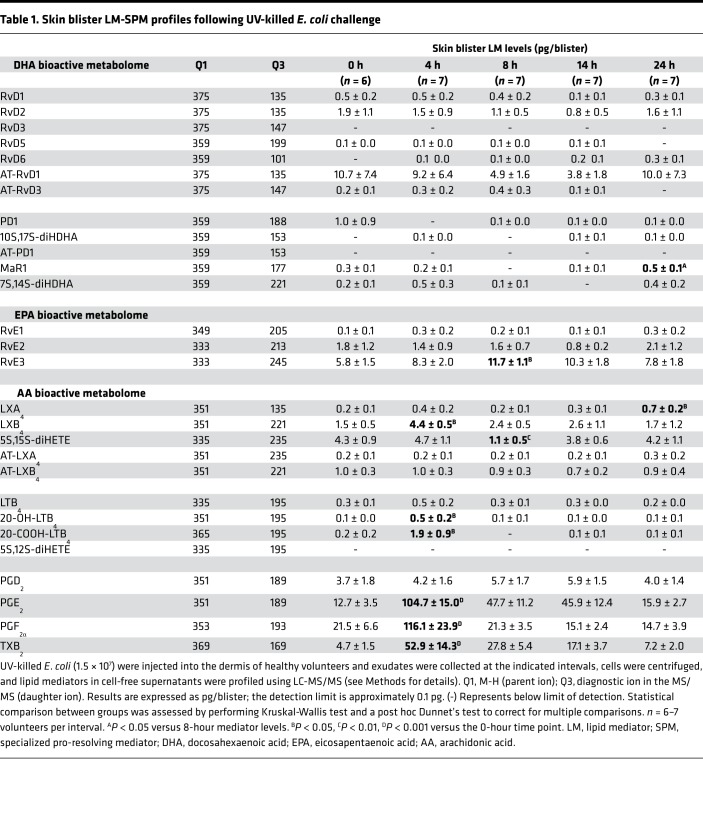
Skin blister LM-SPM profiles following UV-killed *E. coli* challenge

## Version 1 03/22/2018

Electronic publication

## Version 2 07/19/2018

Updated license to Creative Commons.

## Version 3 07/23/2018

The PDF was updated to note the CC BY license.
