# Supplementary material for: Pro-resolving mediators promote resolution in a human skin model of UV-killed Escherichia coli–driven acute inflammation
Source: JCI Insight. 2018 Mar 22;3(6):e94463. doi: 10.1172/jci.insight.94463 (PMC5926908; doi:10.1172/jci.insight.94463)
Supplement: Supplemental data [file jciinsight-3-94463-s009.pdf]

Supplementary data, Figure 1: Integrity of the lipid mediators was assessed using characteristic UV chromophore by spectrophotometry and retention along characteristic MS-MS fragmentation in LC-MS-MS.

AT-RvD1

>99% purity, <1% double bond isomers

180 pg/aliquot

MRM chromatogram of AT-RvD1 standard (m/z 375>215)

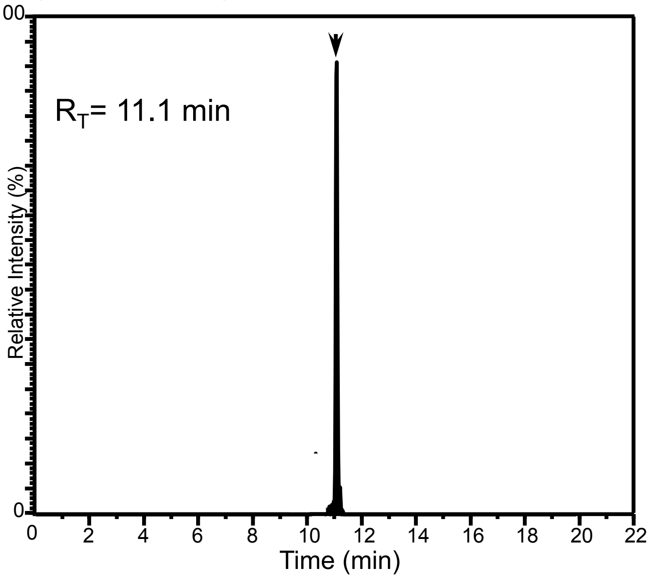

UV Chromophore of AT-RvD1 in methanol

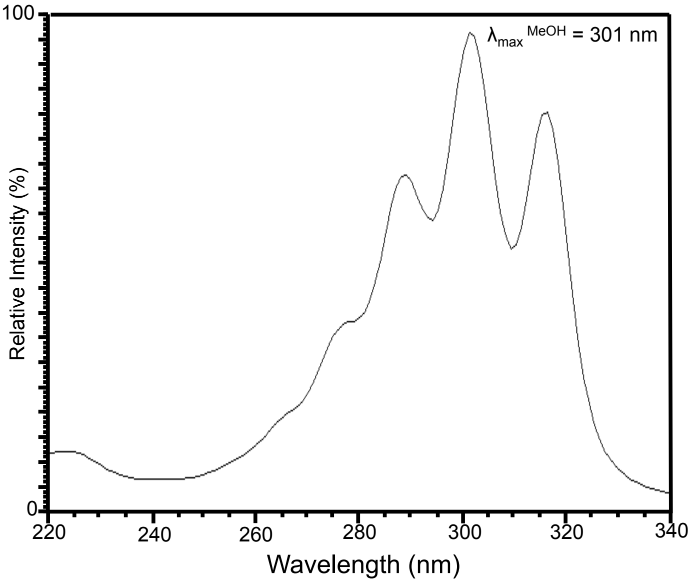

MRM chromatogram of AT-RvD1 (m/z 375>215)

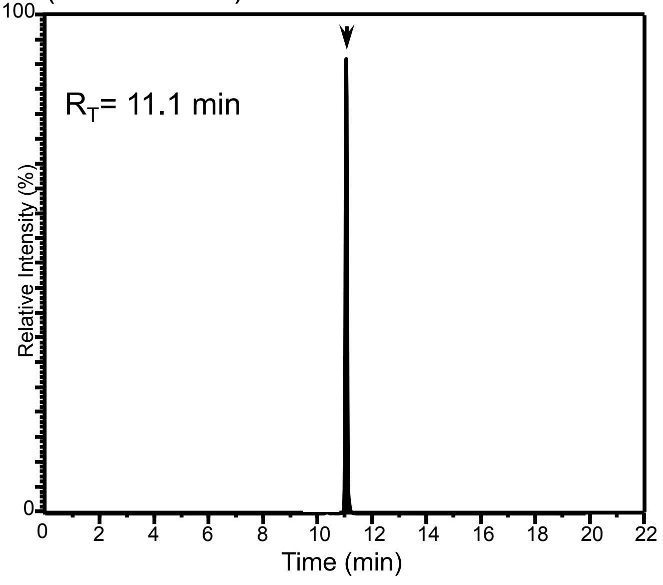

MS-MS Fragmentation spectrum of AT-RvD1

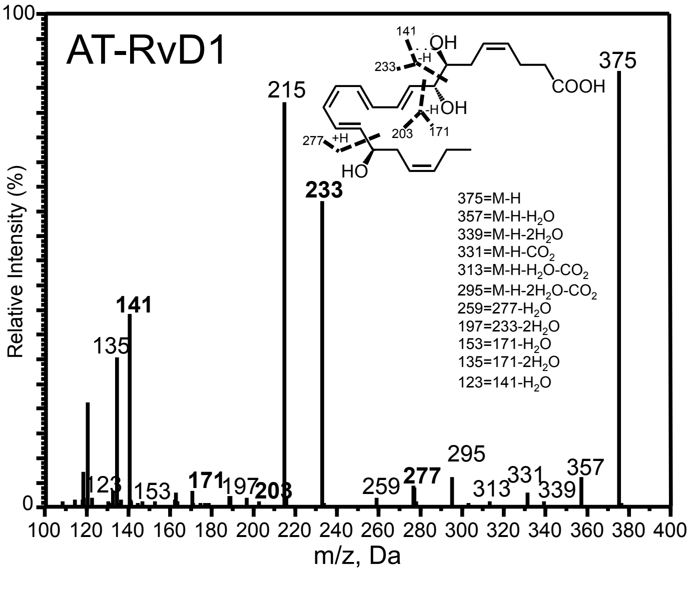

Supplementary data, Figure 1

LXB_4

>99% purity, <1% double bond isomers

30 pg/aliquot

MRM chromatogram of LXB_4 standard (m/z 351>115)

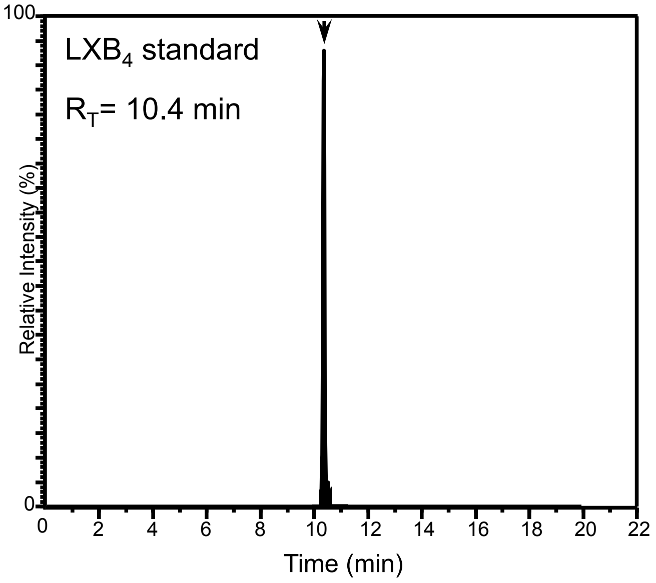

UV Chromophore of LXB_4 in methanol

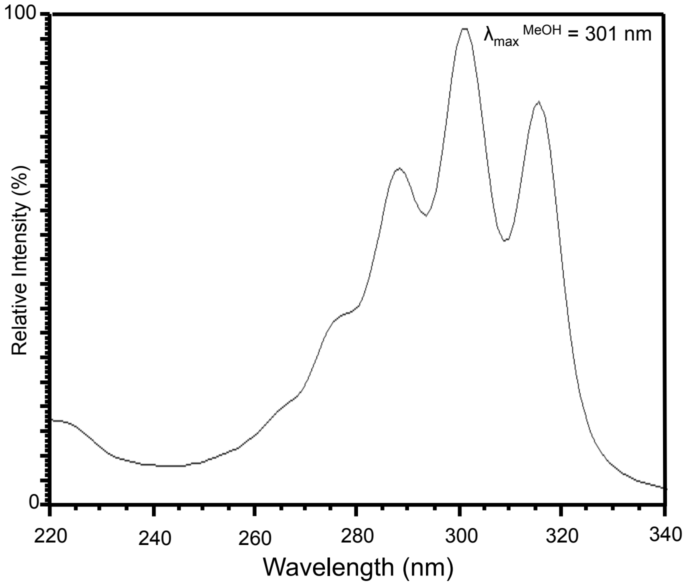

MRM chromatogram of LXB_4 (m/z 351>115)

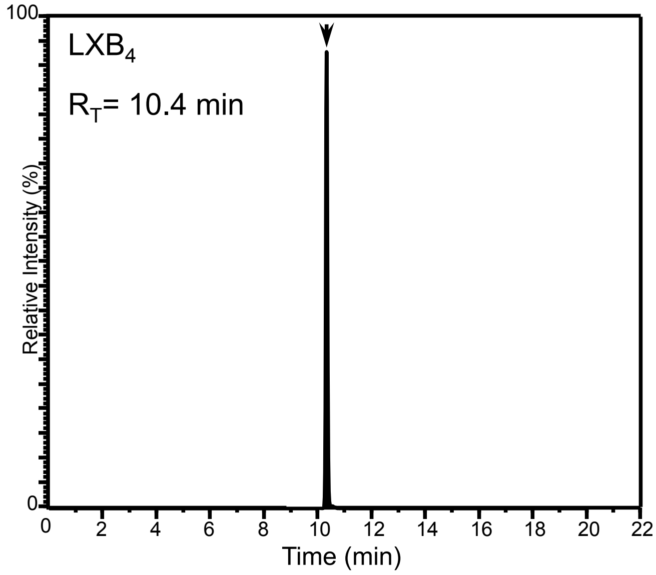

MS-MS Fragmentation spectrum of LXB_4

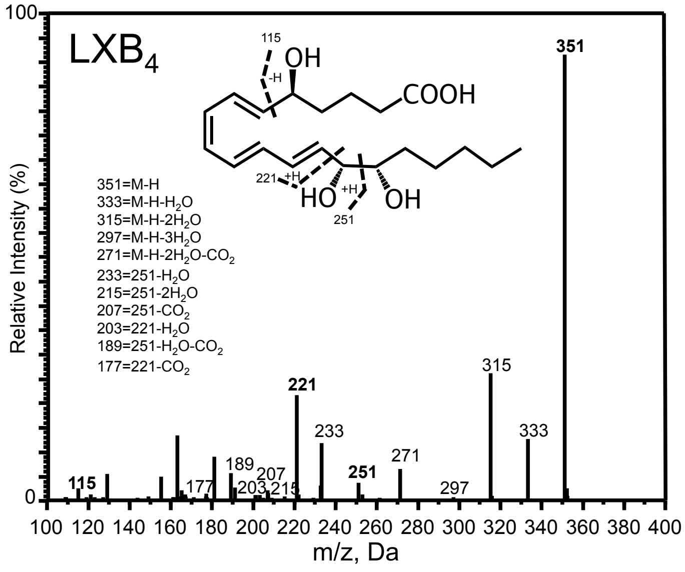

Supplementary data, Figure 1

RvE1

>98% purity, <2% double bond isomers

6 pg/aliquot

MRM chromatogram of RvE1 standard
(m/z 349>161)

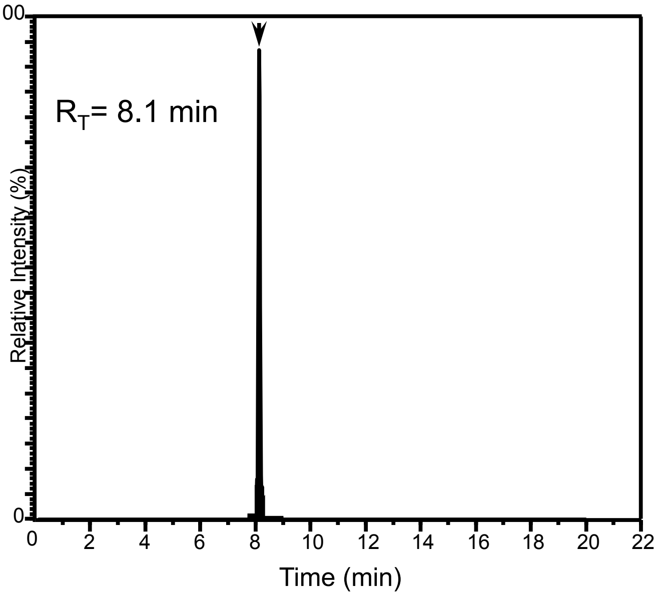

UV Chromophore of RvE1 in methanol

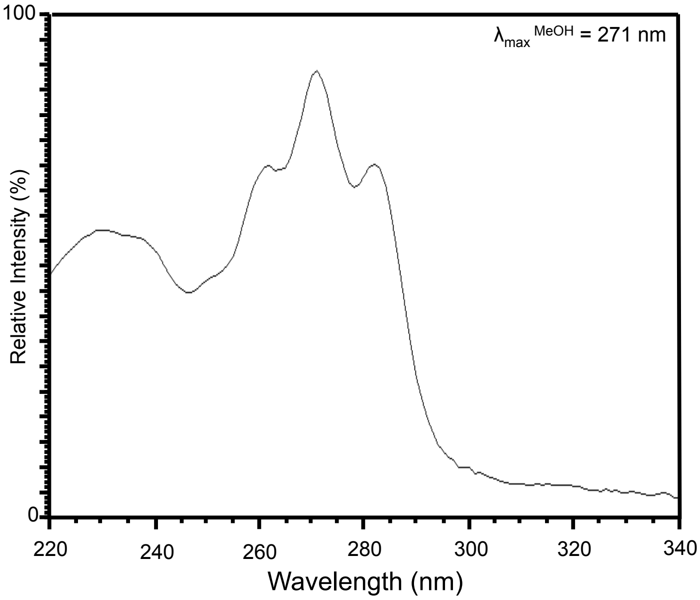

MRM chromatogram of RvE1
(m/z 349>161)

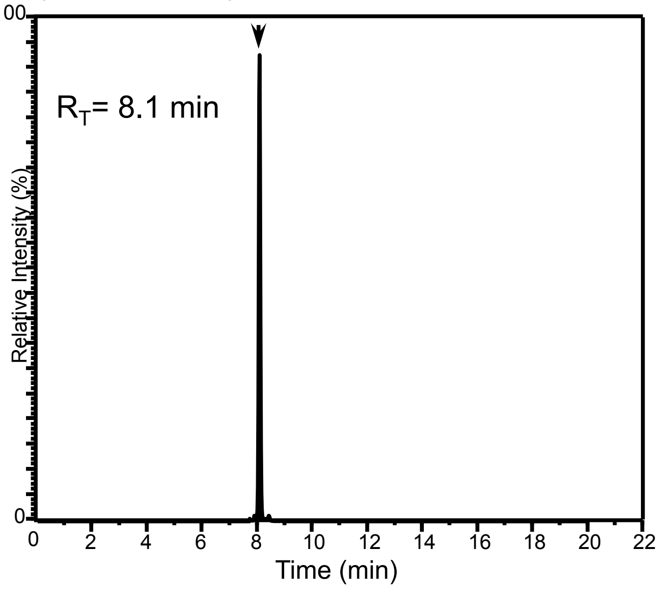

MS-MS Fragmentation spectrum of RvE1

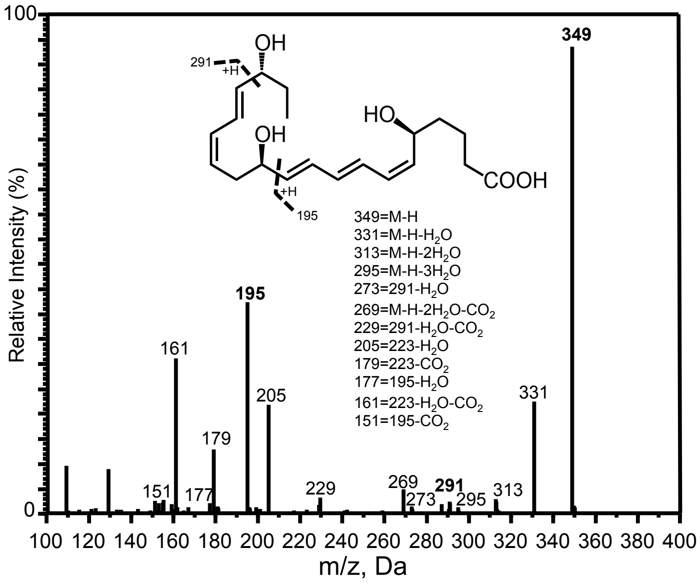

Supplementary data, Figure 1

RvD2

>98% purity, <2% double bond isomers

25.5 pg/aliquot

MRM chromatogram of RvD2 standard (m/z 375>141)

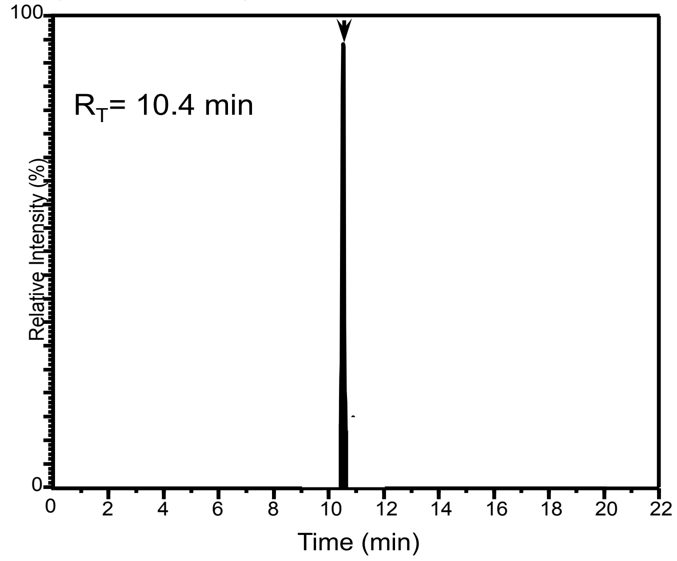

UV Chromophore of RvD2 in methanol

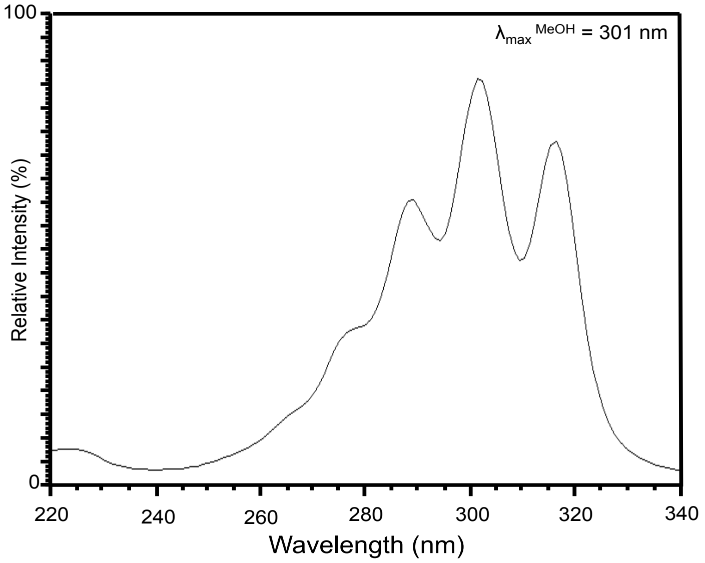

MRM chromatogram of RvD2 (m/z 375>141)

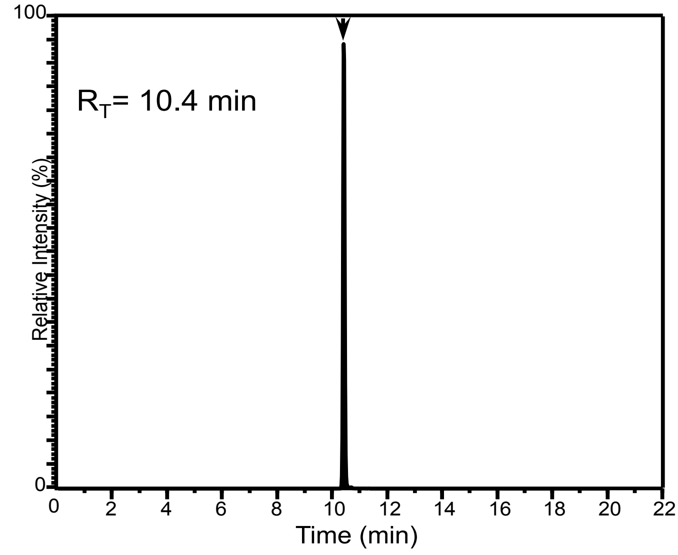

MS-MS Fragmentation spectrum of RvD2

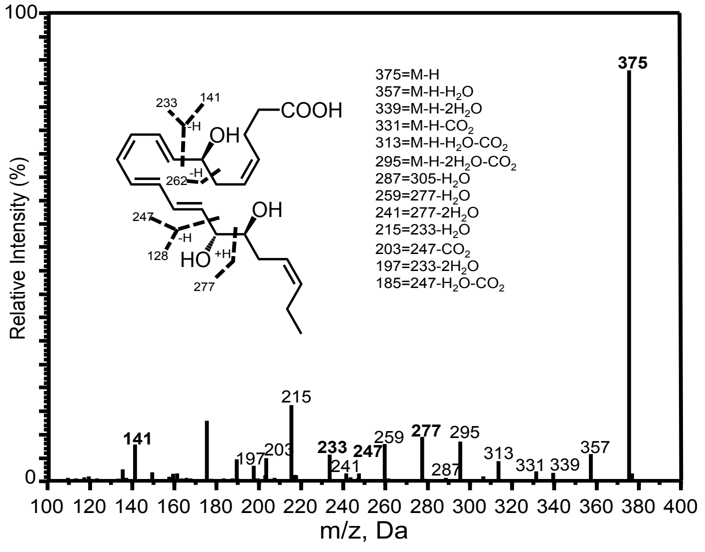

Supplementary data, Figure 2

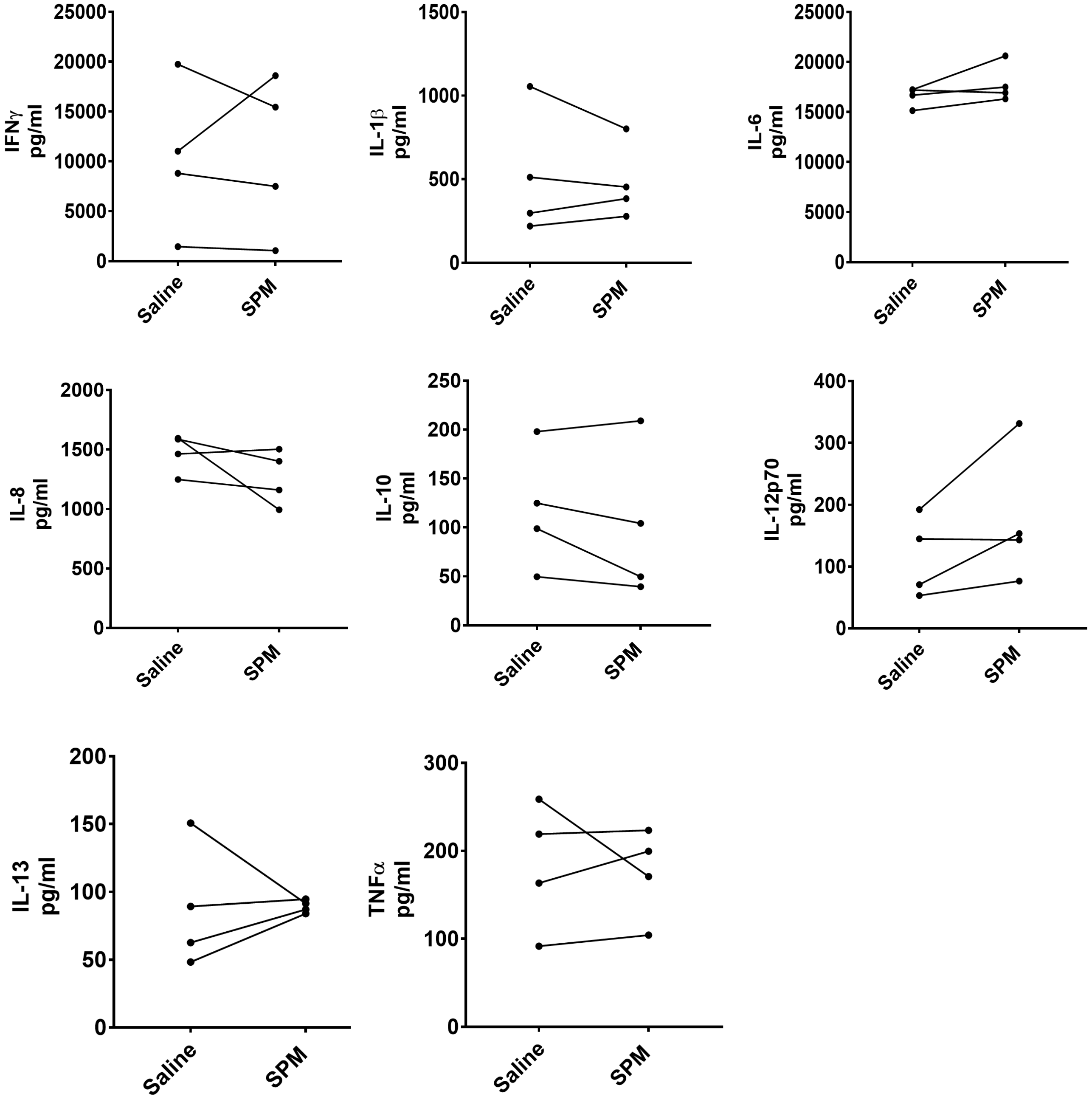

Supplementary data, Figure 2. Actions of SPMs on inflammatory cytokine/chemokine levels in a model of dermal UVKEc-triggered acute inflammation.

Supplementary data, Figure 3

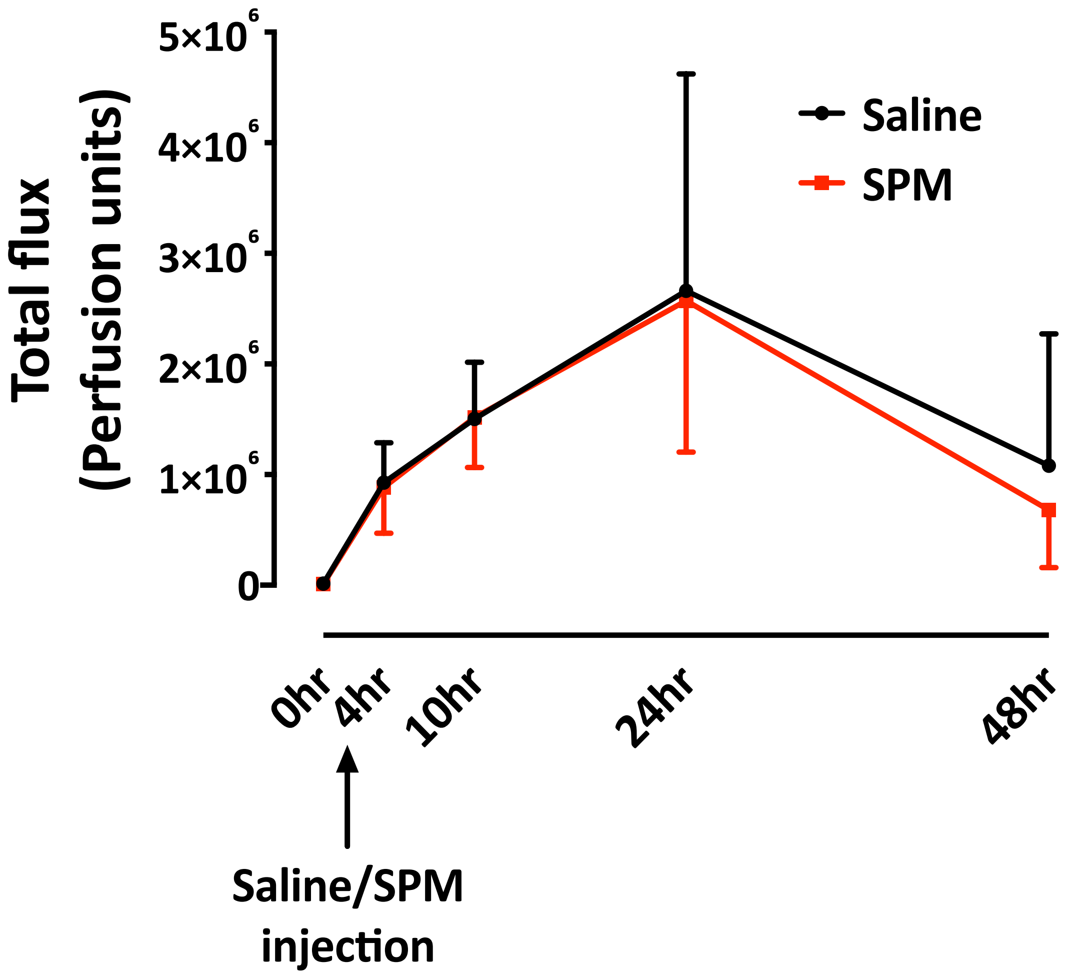

Supplementary data, Figure 3. Effects of SPMs on microvascular hyper reactivity as determined by laser Doppler.
